# Alignment is the Most Efficient Tool in the Management of the Pharmaceutical Turbulent Market

**Published:** 2014

**Authors:** Mahdi Mohammadzadeh

To create values in the working environment and to achieve the organizational aims, organizations have to manage their elements with specific alignment.

In principle, different departments of organizations are put together with specific arrangements and tasks in order to create value for the stakeholders .It has been proven that creating value and the outcome of an organization has a direct relation with the strategic matching among the elements of an organization.

Gordon Jock Churchman (1980) applied system to a group of elements which depend on each other interactively. Although in this definition internal organizational factors are well mentioned, the external effective factors of an organization are not notified. Mehdi Mohammadzadeh (2013) has proved the positive effects of strategic alignment between marketing and financial functions on organizational performance. A system with a thorough definition is divided into two types; Open system and Close system.

The close system does not have interactive relation with external environment of the organization and the internal elements work with each other with a defined procedure but in an open system, the internal elements of the organization not only interact with each other but also they intelligently contact with external environment. In this very type, of which mainly commercial and social issues make benefit, the internal elements of the organization are formed based on taking effects of the environmental factors or the effectiveness of the internal elements.

All parts of a pharmaceutical system from a pharmacy or a retailer to an international manufacturing company with different types of pharmaceutical productions, are known by their open system which has continual interact with their external environment. A pharmacist in a pharmacy not only has to perform his/her job and supply the required medications for the patients, but he/she also must be adequately prepared to give information to the patients and also the other people who live around the pharmacy. In fact this interaction is highly effective on the operation of the pharmacy and its success.

In a manufacturing pharmaceutical company the issue is more complex. In this organization, the institution has to effectively communicate with stakeholders, patients, doctors, pharmacists, investors and Patient Maintenance Organizations and the patient’s advocates, so that they can prepare the beneficial services and production according to their needs and based on their satisfactions. In addition, in order to have a sufficient and efficient operation they should have a constructive interaction.

By a thorough outlook, government and a part of it which supplying medicine falls on, is known as an open system with a wide range of operations. In this system besides the internal elements of the organization that should properly be in an alignment, they also have to establish a fruitful interaction with all the determined organizations such as pharmacies and manufacturing companies. Constructive interaction with a common outlook among the elements of pharmaceutical system and supply chain creates an alignment which the result will be collecting information from the inner layer of the society such as patients and people around the pharmacy and also from the financial, industrial and commercial fields to prepare suitable conditions for the best decision making.

Hence, via this thorough information and analysis of the client’s needs, wants and prediction of their demands promptly, the supplying program would be qualitative, affordable and also accessible.

Thus Iranian Food and Drug Organization can establish a program for constructive alignment between different elements of pharmaceutical system to manage pharmaceutical market and provide the patient’s needs in unstable situations.

*Mahdi Mohammadzadeh is currently working as assistant Professor and Head of Department of Pharmacoeconomic, School of Pharmacy, Shahid Beheshti University of Medical Sciences, Tehran, Iran. He could be reached at the following e-mail address: mohammadzadeh655@yahoo.com*

